# Characteristics of Tianeptine Exposures Reported to the National Poison Data System — United States, 2000–2017

**DOI:** 10.15585/mmwr.mm6730a2

**Published:** 2018-08-03

**Authors:** Tharwat El Zahran, Joshua Schier, Emily Glidden, Stephanie Kieszak, Royal Law, Edward Bottei, Cynthia Aaron, Andrew King, Arthur Chang

**Affiliations:** ^1^Division of Environmental Health Science and Practice, National Center for Environmental Health, CDC; ^2^Emory University School of Medicine, Atlanta, Georgia; ^3^Iowa Poison Control Center, Sioux City, Iowa; ^4^Children’s Hospital of Michigan Regional Poison Control Center, Detroit.

Tianeptine (marketed as Coaxil or Stablon) is an atypical tricyclic drug used as an antidepressant in Europe, Asia, and Latin America. In the United States, tianeptine is not approved by the Food and Drug Administration (FDA) for medical use and is an unscheduled pharmaceutical agent* (*1*). Animal and human studies show that tianeptine is an opioid receptor agonist (*2*). Several case studies have reported severe adverse effects and even death from recreational abuse of tianeptine (*3*–*5*). To characterize tianeptine exposures in the United States, CDC analyzed all exposure calls related to tianeptine reported by poison control centers to the National Poison Data System (NPDS)^†^ during 2000–2017. Tianeptine exposure calls, including those for intentional abuse or misuse, increased across the United States during 2014–2017, suggesting a possible emerging public health risk. Most tianeptine exposures occurred among persons aged 21–40 years and resulted in moderate outcomes. Neurologic, cardiovascular, and gastrointestinal signs and symptoms were the most commonly reported health effects, with some effects mimicking opioid toxicity. A substantial number of tianeptine exposure calls also reported clinical effects of withdrawal. Among 83 tianeptine exposures with noted coexposures, the most commonly reported coexposures were to phenibut, ethanol, benzodiazepines, and opioids.

CDC used NPDS data to review all tianeptine exposure telephone calls reported by U.S. poison control centers during 2000–2017. Calls for drug information or identification were excluded. Trends in exposure by year were compiled for all tianeptine exposure calls and for calls related to intentional abuse or misuse. Descriptive statistics were compiled for all exposure calls by U.S. Census region (Midwest, Northeast, South, and West),^§^ caller source (self or health care professional), demographics (sex and age group), exposure type (intentional, unintentional, withdrawal, or unknown/other), exposure route (ingestion, parenteral, or inhalation), and coexposures. Tianeptine-only exposures were defined as those that reported only tianeptine use with no other substances. Tianeptine-only exposures (excluding withdrawal-associated calls) were analyzed for reported related clinical effects by body systems, performed therapies, and level of care (evaluated, treated, and released from the emergency department (ED), admission to noncritical care units, admission to critical care units, or other).

Exposure medical outcomes were classified according to American Association of Poison Control Centers (https://www.aapcc.org/) definitions as either no effect, minor outcome, moderate outcome, severe outcome, or death. Minor outcomes were defined as symptoms that were minimally bothersome to the patient, usually resolved rapidly, and often involved skin or mucous membrane manifestations, after which the patient returned to a preexposure state of well-being with no residual disability or disfigurement. Moderate outcomes were defined as symptoms that were more pronounced, more prolonged, or of a more systemic nature than minor symptoms but were not life threatening; usually requiring some form of treatment, after which the patient returned to a preexposure state of well-being with no residual disability or disfigurement. Major outcomes were defined as symptoms that were life threatening or resulted in a significant residual disability or disfigurement. Withdrawal-associated tianeptine calls were analyzed separately for clinical effects and performed therapies. Summaries of two cases reported in 2016 are presented for illustrative purposes (Supplementary Table, https://stacks.cdc.gov/view/cdc/57404).

Frequencies for categorical variables and mean values for continuous variables were calculated using statistical software. Tests for the trend for all tianeptine exposure calls and for calls related to intentional abuse or misuse during 2014–2017 were performed. Fisher’s exact test was used to test for associations between reported outcome severity and tianeptine-only exposures versus tianeptine with coexposures, age group, and sex. Statistical significance was defined as p<0.05.

During 2000–2017, NPDS received 218 calls related to tianeptine exposure, including one from outside the United States. Tianeptine-only exposures, excluding 29 withdrawal-associated calls, accounted for 114 (52.3%) calls. During the first 14 years of the study period (2000–2013), NPDS received a total of 11 tianeptine exposure calls. From 2014 through 2017, there was a statistically significant increase in calls related to exposure (p<0.001) and intentional abuse or misuse (p<0.001). The total number of tianeptine exposure calls increased from five in 2014 to 38 in 2015, 83 in 2016, and 81 in 2017 (Figure). The majority of calls (91.2%) came from health care providers; by U.S. Census region, the highest percentage of calls came from the South (34.6%). Among 213 (97.7%) exposure calls for which information on age was available, the mean age was 35 years (range = 1–80 years) (Table 1).

**FIGURE:**
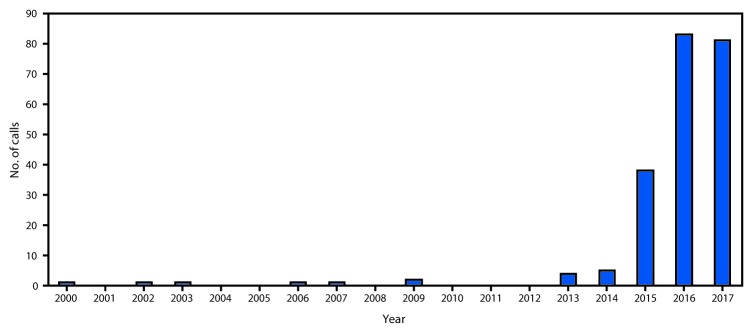
Number of tianeptine exposure telephone calls reported (N = 218) — National Poison Data System, United States, 2000–2017

**TABLE 1:** Characteristics of telephone calls related to tianeptine exposure (N = 218) — National Poison Data System, United States, 2000–2017

Characteristic (no. with known information)	No.	(%)
**Call source (218)**		
Health care provider	198	(91.2)
Caller residence	13	(6.0)
Other	7	(3.2)
**U.S. Census region (217)**		
South	75	(34.6)
West	54	(24.9)
Midwest	47	(21.6)
Northeast	41	(18.9)
**Sex (215)**		
Male	177	(82.3)
Female	38	(17.7)
**Age group (yrs) (213)**		
<20	25	(11.7)
21–40	121	(56.8)
41–60	59	(27.7)
≥61	8	(3.8)
**Exposure route (218)**		
Ingestion	183	(83.9)
Parenteral	15	(6.9)
Inhalation	4	(1.8)
Unknown/Other	16	(7.4)
**Exposure type (218)**		
Intentional	119	(54.6)
Unintentional	23	(10.5)
Withdrawal	29	(13.3)
Unknown/Other	47	(21.6)
**Coexposure (83)**		
Phenibut	26	(31.3)
Ethanol	13	(15.7)
Benzodiazepines	10	(12.0)
Opioids	10	(12.0)

Among the 114 tianeptine-only exposures, excluding withdrawal-related calls, the most commonly reported related clinical effects were neurologic (48.3%), cardiovascular (32.5%), and gastrointestinal (10.5%) (Table 2). The most commonly administered therapies included fluids (35.1%), benzodiazepines (27.2%), and oxygen (10.5%) (Table 2). Among the 105 exposure calls for which level of care was reported, 46 (44%) persons were treated, evaluated, and released from the ED, and 25 (24%) were admitted to a critical care unit. Among the 93 tianeptine-only exposures with a known medical outcome, 50 (54%) had moderate outcomes. No deaths were reported.

**TABLE 2:** Common clinical effects associated with tianeptine exposures (N = 114**) **and therapies received — National Poison Data System, United States, 2000–2017

Clinical effect*	No.	(%)
**Cardiovascular effect**	**37**	**(32.5)**
Tachycardia	29	(25.4)
High blood pressure	13	(11.4)
Conduction delays	5	(4.4)
**Neurologic effect**	**55**	**(48.3)**
Agitation	25	(21.9)
Drowsiness	19	(16.7)
Confusion	15	(13.2)
Coma	5	(4.4)
**Gastrointestinal effect**	**12**	**(10.5)**
Nausea	9	(7.9)
Vomiting	5	(4.4)
Diarrhea	3	(2.6)
**Dermal effect**	**10**	**(8.8)**
Pallor	3	(2.6)
Pain	3	(2.6)
Cellulitis	2	(1.8)
**Constitutional effect**	**10**	**(8.8)**
Diaphoresis	8	(7.0)
Fever	3	(2.6)
Pain	1	(0.9)
**Respiratory effect**	**8**	**(7.0)**
Respiratory depression	6	(5.3)
Dyspnea	3	(2.6)
Tachypnea	1	(0.9)
**Ocular effect**	**6**	**(5.3)**
Mydriasis	4	(3.5)
Miosis	2	(1.8)
**Renal effect**	**5**	**(4.4)**
Urinary retention	3	(2.6)
Creatinine abnormality	2	(1.8)
Kidney failure	1	(0.9)
**Metabolic effect**	**5**	**(4.4)**
Electrolyte disturbances	3	(2.6)
Acidosis	2	(1.8)
**Musculoskeletal effect**	**5**	**(4.4)**
Muscle weakness	2	(1.8)
Rigidity	1	(0.9)
**Psychiatric effect**	**2**	**(1.8)**
Delusions	2	(1.8)
**Therapy**		
Fluids	40	(35.1)
Benzodiazepines	31	(27.2)
Oxygen	12	(10.5)
Naloxone	11	(9.7)
Antibiotics	11	(9.7)
Sedation	9	(7.9)
Antiemetics	7	(6.1)
Intubation	5	(4.4)
Ventilator support	5	(4.4)
Antihistamine	3	(2.6)

* Patient exhibited one or more type of clinical effect in a category.

Poison control centers reported 29 withdrawal-associated calls. Among those, tianeptine was the only substance reported to be associated with withdrawal in 21 (72.4%) calls; among those 21 calls, the most frequently reported signs and symptoms were agitation (33.3%), nausea (33.3%), vomiting (19%), tachycardia (19.1%), hypertension (14.3%), diarrhea (9.5%), tremor (9.5%), and diaphoresis (9.5%). The most frequently administered therapies included benzodiazepines (57.1%), fluids (38.1%), and antiemetics (19.1%).

Among the 183 exposure calls with a known outcome, significant associations were found between outcome severity for tianeptine-only exposures versus tianeptine with coexposures (p = 0.01) and between outcome severity and sex (p = 0.02). Persons reporting coexposure along with tianeptine were more likely to have major outcomes than those with tianeptine-only exposures. Men were more likely than women to have a moderate outcome. No differences were found between outcome severity and age group (<20, 21–40, 41–60, and ≥61 years) (p = 0.93).

## Discussion

This study revealed a nationwide increase in tianeptine exposure calls and calls related to intentional abuse and misuse during 2014–2017. Approximately half of all reported exposures occurred among users aged 21–40 years. The increase in exposures from 2014 to 2017 might be explained by a 2014 study in animals and humans that showed that tianeptine is an effective mu- and delta-opioid receptor agonist (*2*). Deaths associated with misuse of tianeptine have been reported outside the United States (*3*,*4*). Recently, two deaths that were not reported to NPDS during the study period and were attributed to tianeptine toxicity were reported in the United States in persons who purchased the drug online (*5*).

Several case reports showed that tianeptine toxicity mimicked opioid toxicity and that naloxone was an effective therapy (*6*,*7*). Tolerance to tianeptine and withdrawal have been reported (*8*). Neonatal abstinence syndrome mimicking opioid neonatal abstinence syndrome has occurred after tianeptine dependence during pregnancy (*9*). This study further highlights that the withdrawal effects of tianeptine mimic those of opioid withdrawal.

Tianeptine has an abuse potential in former opiate drug users (*3*). In the country of Georgia, the health authority withdrew tianeptine from the market in June 2010, and the health authorities of Russia and Armenia classified tianeptine as a controlled substance in July 2010 (*3*). Similar measures were implemented in Ukraine in January 2011 (*3*). Although tianeptine is not FDA approved in the United States, it is readily available for purchase online as a dietary supplement or research chemical. Several online discussion forums among users describe the euphoregenic effects of tianeptine. Users have also reported combining tianeptine with other drugs like phenibut for a potentiated effect. In this study, among 83 calls with reported coexposures, phenibut (26 [31%]) was the most commonly reported coexposure with tianeptine. In light of the ongoing U.S. opioid epidemic, any emerging trends in drugs with opioid-like effects raise concerns about potential abuse and public health safety.

The findings in this report are subject to at least three limitations. First, NPDS relies on data voluntarily reported to poison control centers by health care providers and the public, who might not have reported all tianeptine exposures to poison control centers. Second, unintentional coding errors could have occurred during documentation. Finally, NPDS data provide only limited clinical information. For example, information on treatment response, length of hospital stay, or residual sequelae are not available.

This analysis highlights recent increases in reported tianeptine use and the potential for abuse and effects associated with withdrawal that can make it difficult to reduce or discontinue use. The associated outcomes and health effects associated with tianeptine use suggest a possible emerging public health risk and underscore the need for public outreach to increase awareness. Tianeptine testing is not routinely available, but specialty-testing laboratories might have that capacity. Health care providers and public health officials need to be vigilant for potential cases of tianeptine exposure and, when applicable, report adverse effects to the FDA MedWatch reporting system (https://www.fda.gov/Safety/MedWatch/default.htm). Clinicians and other health care providers can contact their local poison control center by telephone at 1-800-222-1222 for clinical guidance as needed.

**Summary**

**What is already known about this topic?**

Tianeptine is an antidepressant drug that is not approved by the Food and Drug Administration (FDA). Clinical effects of tianeptine abuse and withdrawal can mimic opioid toxicity and withdrawal.

**What is added by this report?**

Tianeptine exposure calls to U.S. poison control centers increased during 2014–2017, suggesting a possible emerging public health risk. The associated health effects included neurologic, cardiovascular, and gastrointestinal signs and symptoms, with some effects mimicking opioid toxicity and withdrawal.

**What are the implications for public health practice?**

Health care provider and public education about adverse effects associated with tianeptine use is warranted. Health care providers and public health officials need to report adverse effects to the FDA MedWatch reporting system and contact poison control centers for clinical guidance.

## Footnotes

† NPDS is a national database of information provided by the country’s regional poison centers serving all 50 states, the District of Columbia, the U.S. Virgin Islands, and Puerto Rico. The American Association of Poison Control Centers maintains the database. NPDS case records are the result of call reports made by members of the public or health care providers. https://www.aapcc.org/data-system/.

§ https://www.census.gov/geo/reference/webatlas/regions.html.
